# Integrating artificial intelligence with genome sequencing against antimicrobial resistance: a narrative review

**DOI:** 10.3389/fpubh.2026.1757161

**Published:** 2026-01-29

**Authors:** Giovanni Scaglione, Nicolò Mastroianni, Alberto Rizzo, Emanuele Palomba, Davide Carcione, Sara Giordana Rimoldi, Gioconda Brigante, Luigi Principe, Marta Colaneri, Andrea Gori, Fabio Borgonovo

**Affiliations:** 1 Department of Infectious Diseases, Luigi Sacco Hospital, Milan, Italy; 2 Laboratory of Clinical Microbiology and Virology, ASST Valle Olona, Gallarate, Italy; 3 Laboratory of Clinical Microbiology, Virology and Bioemergencies, Luigi Sacco Hospital, Milan, Italy; 4 Centre for Multidisciplinary Research in Health Science (MACH), University of Milan, Milan, Italy; 5 Clinical Microbiology and Virology Unit, Great Metropolitan Hospital "Bianchi-Melacrino-Morelli", Reggio Calabria, Italy; 6 Department of Biomedical and Clinical Sciences "L. Sacco", University of Milan, Milan, Italy

**Keywords:** antimicrobial resistance, artificial intelligence, diagnosis, genome sequencing, infection, machine learning, surveillance

## Abstract

Antimicrobial resistance (AMR) represents an escalating global health threat, demanding diagnostic strategies capable of rapid, accurate, and comprehensive pathogen characterization. Genomic sequencing has transformed our ability to elucidate resistance mechanisms and track their evolution, yet its routine clinical adoption remains limited by cost, workflow constraints, and extended turnaround times. This narrative review examines how artificial intelligence (AI) and machine learning (ML) can enhance and operationalize sequencing-based diagnostics across the clinical microbiology continuum. We summarize current AI applications in whole-genome sequencing for AMR prediction, pan-genome feature extraction, and multicenter model generalizability, including emerging approaches such as federated learning. We then explore AI-driven metagenomic analytics for pathogen detection, resistome profiling, outbreak investigation, and prognostic modeling. Complementary non-genomic technologies, Raman spectroscopy and MALDI-TOF MS, are also evaluated for their potential to deliver rapid resistance profiling when integrated with ML. Finally, we discuss practical barriers, including cost, dataset standardization, interpretability, and regulatory challenges, while outlining future directions toward scalable, explainable, and equitable AI-guided diagnostics. Integrating AI with genomic and rapid phenotypic tools offers a pathway to real-time surveillance, optimized antimicrobial stewardship, and strengthened preparedness against emerging infectious threats.

## 1 Introduction

Antimicrobial resistance (AMR) is rapidly becoming a major global health threat with the World Bank estimating that AMR could result in US$ 1 trillion additional healthcare costs by 2050, and US$ 1–3.4 trillion gross domestic product losses per year by 2030. It has been reported that poor access to quality diagnostics is one of the main contributing factors, among others (1). The advent of genomic sequencing has significantly enhanced our ability to understand the genetic mechanisms behind resistance, track their evolution over time, and accurately monitor the spread of resistant strains.

However, despite its analytical power, the integration of genome sequencing into routine clinical workflows remains limited. Barriers such as cost, personnel training requirements, and longer turnaround time (TAT) compared to traditional phenotypic testing or polymerase chain reaction (PCR) assays continue to delay its broader adoption (2).

In parallel, artificial intelligence (AI) has been proposed as a revolutionizing tool for laboratory sample processing and data management. Specifically, machine learning (ML) systems, with the ability to learn and improve from training datasets, are emerging with a central role in clinical microbiology (3). Beyond ML, more sophisticated AI frameworks, including convolutional neural networks (CNNs) and large language models (LLMs) are being investigated for their ability to handle complex, high-dimensional datasets and generate clinically relevant outputs (4–6).

Recent studies suggest that AI may play an integrative role across the full continuum of pathogen genomics, from raw data processing to the generation of actionable insights, potentially addressing several obstacles that limit the scalability of sequencing-based diagnostics (7). Here, we examine how AI-driven approaches can enhance genomic surveillance, resistance prediction, and the broader fight against AMR when strategically combined with next-generation sequencing (NGS) and complementary technologies.

## 2 Approach and scope of the narrative review

This narrative review was conducted to synthesize and critically appraise current evidence on the integration of AI with genomic and rapid diagnostic technologies for AMR detection and surveillance in hospital and public health settings. Abbreviations, and the corresponding brief definitions, of all AI/ML-related terms used in this manuscript are provided in Table 1. The review was informed by a predefined conceptual PI(C)O framework (Supplementary Table S1), which served as a scoping scaffold to define the population, intervention domains, and outcomes of interest, rather than as a formal systematic or scoping review protocol. The literature search was conducted across three major biomedical databases: MEDLINE (via Ovid), Embase, and the Cochrane Central Register of Controlled Trials (CENTRAL). Searches included publications up to April 2025, with particular emphasis on studies published within the last decade, reflecting the rapid evolution of AI methodologies and sequencing technologies. Search concepts combined terms related to AI and ML (e.g., “AI,” “machine learning,” “deep learning,” “natural language processing”), genomic and rapid diagnostic technologies (e.g., “next-generation sequencing,” “whole-genome sequencing,” “metagenomics,” “nanopore sequencing,” “MALDI-TOF MS,” “Raman spectroscopy”), AMR, and surveillance or infection prevention workflows.

**Table 1:** Abbreviations and brief definitions of AI/ML-related terms used in the manuscript.

Abbreviation	Extended name	Brief definition
AI	Artificial Intelligence	Umbrella term for computational systems designed to perform tasks requiring human-like intelligence, including learning, reasoning, and decision-making.
ML	Machine Learning	Subfield of AI in which algorithms learn patterns from data to make predictions or decisions without explicit rule-based programming.
DL	Deep Learning	Subset of ML using multi-layered neural networks to model complex, high-dimensional relationships in data.
CNN	Convolutional Neural Network	DL architecture optimized for grid-like data (e.g., spectra, signals, images), using convolutional filters to learn local patterns.
ANN	Artificial Neural Network	ML model inspired by biological neurons, consisting of interconnected layers that transform inputs into predictive outputs.
DT	Decision Tree	Supervised ML model that recursively splits data using decision rules to predict outcomes.
RF	Random Forest	Ensemble ML method combining multiple decision trees trained on random feature subsets to improve predictive accuracy and robustness.
SVM	Support Vector Machine	Supervised ML algorithm that finds optimal separating hyperplanes to classify or regress high-dimensional data.
LR	Logistic Regression	Supervised statistical-ML model estimating the probability of a binary outcome using a logistic function.
XAI	Explainable Artificial Intelligence	Methods that provide transparent, interpretable explanations of AI model predictions to support trust and auditability.
k-mer	k-length nucleotide substring	Fixed-length sequence fragments used as alignment-free genomic features in ML models.

The scope of this review is intentionally restricted to AI applications integrated with genomic and rapid diagnostic technologies for AMR detection, characterization, and surveillance. It does not aim to provide a comprehensive review of AI tools focused primarily on patient-level MDRO carrier prediction, hospital logistics, or infection control management, which fall outside the focus of the present manuscript.

The review prioritized studies relevant to human health, particularly those addressing hospital or inpatient settings, population-level surveillance, infection prevention and control (IPC), and antimicrobial stewardship. One Health or environmental studies were included when they directly informed surveillance frameworks, transmission dynamics, or public health implementation. We explicitly excluded studies limited to: (i) AI applications focused solely on antimicrobial prescribing optimization or laboratory automation without infection control relevance, and (ii) theoretical models, simulations, or proof-of-concept frameworks lacking a clear pathway to real-world application.

Priority was given to multicenter studies, externally validated models, real-world or near-real-world implementations, and consensus documents or guidelines where available. Single-center and early proof-of-concept studies were included selectively to illustrate emerging approaches, but were interpreted cautiously with respect to generalizability, validation, and readiness for implementation. Up-to-date narrative reviews were included to enhance the completeness of the literature coverage. During manuscript revision, nine relevant articles published after April 2025 were identified and incorporated as substantive updates in the field, and four studies initially excluded were subsequently included following re-evaluation of their relevance.

This review is intentionally narrative and non-exhaustive. No formal systematic review methodology, risk-of-bias assessment, or quantitative synthesis was undertaken. The objective was to provide a structured and critical overview of key AI-enabled genomic and rapid diagnostic applications for AMR surveillance, highlighting methodological limitations, operational constraints, and implications for public health practice.

## 3 Artificial intelligence and genome sequencing

The critical threat of AMR is prompting the need for advanced computational methods capable of rapid and precise resistance prediction. Recent developments in ML leverage genomic data, pan-genome analyses, and ensemble techniques to enhance detection accuracy. Nevertheless, challenges persist in feature selection, model generalizability, and validation across heterogeneous datasets.

A foundational step in AMR prediction is identifying informative genomic features. Traditional models often rely on known resistance genes or mutations, which may overlook novel or combinatorial signals. Her and Wu demonstrated that pan-genome-based feature extraction improved prediction accuracy in *Escherichia coli* by capturing accessory genome elements linked to strain-specific resistance mechanisms (8). Notably, non-core genes, present only in subsets of strains, outperformed core-genome features, even though not all directly conferred resistance. Many of these genes, in fact, often carried on mobile elements like plasmids, contributed to broader multidrug resistance signals. The proposed AI-based genetic algorithm approach based on non-core genes identified informative clusters that discriminated resistant from susceptible *E. coli*, achieving area under the curve (AUCs) above 0.9 for ampicillin, gentamicin, trimethoprim-sulfamethoxazole, and ciprofloxacin, surpassing conventionally examined clusters. Notably, the model flagged the *pmrC/pmrE* cluster (classically associated with polymyxin resistance) as predictive not only of polymyxin resistance but also of ampicillin and trimethoprim–sulfamethoxazole resistance, underscoring the capacity of ML to reveal latent resistance patterns.

Similarly, Wang et al. (9) showed that combining ML with whole-genome sequencing (WGS) predicted *Staphylococcus aureus* minimum inhibitory concentrations with >95% accuracy, particularly excelling in detecting cefoxitin resistance and reliably identifying methicillin-resistant *S. aureus* (MRSA) strains.

In this context Hyun et al. (10) developed a pan-genome framework for *S. aureus*, *P. aeruginosa*, and *Escherichia coli*, creating a binary genome-by-feature matrix to train multiple support vector machines (SVM) models. Their ensemble approach, based on randomly sampled strain-feature subsets, achieved accuracies between 79.3 and 99.5%, outperforming classical statistical and ranking methods. Impressively, the model recovered 45 known resistance genes and suggested 25 novel candidates (10). By training every sub model on a randomly selected part of the dataset, they reduced overfitting and improved model generalizability.

Álvarez et al. (11), for instance, applied SVM to 5,299 *A. baumannii* genomes, including 512 global clone 1 (GC1) and 4,787 non-GC1, and identified a previously unknown 367 base pair (bp) fragment of *moaCB*, termed U1, uniquely defining the high-risk GC1. The model achieved notable performance (sensitivity 1.00, specificity 1.00, accuracy 1.00; 0 false positives and 0 false negatives), illustrating how ML can reveal clinically actionable biomarkers and directly support rapid diagnostic development in outbreak scenarios (11).

Most studies validate on single datasets, risking inflated performance estimates. When this is not the case, model performance is not consistent across different settings, and often fails to replicate across hospitals, likely due to differences in sequencing platforms, population structure, and metadata quality. On the other hand, while multicenter datasets enhance robustness, they also introduce new biases from heterogeneous lab protocols. Federated learning presents a promising alternative, allowing multi-institutional training without sharing raw genomic data. This approach can balance generalizability with privacy, laying the foundation for regional ML-based AMR early-warning systems (12).

Future models may benefit from integrating multi-omics layers, such as transcriptomics or proteomics, to uncover complex interactions or cryptic features that lead to AMR.

## 4 Artificial intelligence and metagenomics

Thanks to rapid advancements in sequencing technologies, metagenomics has the potential to provide a detailed view of the entire microbial ecosystem within clinical and environmental samples. Metagenomics provides a panoramic, high-resolution snapshot of the entire microbial genomic landscape, representing a substantial advance in microbiological diagnostics by moving beyond the targeted detection of individual pathogens and capturing comprehensive genomic information even in the absence of culture growth.

For example, Fida et al. (13) observed that 16S rRNA gene metagenomics was able to identify a potentially pathogenic organism in 47% of septic patients compared to 32% with blood cultures. As sequencing technologies continue to advance, this approach may potentially bypass traditional methods in the future, beginning with direct nucleic acid extraction from the collected specimen.

These technologies, like shotgun metagenomic sequencing, generate massive volumes of raw genetic data. Therefore, scaling this approach requires sequencing and data analysis technologies capable of managing the complexity and volume of information generated (14).

Hence, AI-based data management could be of paramount importance since the task of assembling millions of short reads into a coherent biological narrative is expected to be very time consuming without advanced computational pipelines. AI algorithms are used not only to align reads with reference databases, but more importantly, to perform deeper reconstruction and inference tasks. ML algorithms, specifically deep learning (DL) methods like CNN, could address several aspects of metagenomics sequencing, including microbial population analysis, novel pathogen detection, sequence classification, patient stratification and disease prediction (15).

Additionally, they have been successfully applied to functional annotation, generating comprehensive taxonomic profiles, classifying whether a given sequence is an antibiotic resistance gene, identifying all resistance determinants that together compose the sample’s resistome, and detecting virulence genes or metabolic biomarkers (16, 17).

This broader, systems-level, insight can redefine how antimicrobial resistance is understood and monitored. While conventional methods profile resistance at the level of individual isolates, metagenomics unveils the resistome embedded within a sample’s microbiota, including silent or subdominant reservoirs of resistance that may be mobilized under antibiotic pressure (18). This capacity is especially valuable for colonization screening, where characterizing the full spectrum of resistance genes enables more informed infection control and outbreak prevention strategies.

In a 2025 study, Mikolas et al. (19), through shotgun sequencing on Illumina platform, applied high-throughput metagenomic analysis to 96 intensive care unit (ICU) patient, staff, and environmental samples, aiming to identify early microbial biomarkers for prognostic assessment and uncovered extensive AMR reservoirs. Their computational models identified early oropharyngeal resistome signatures that discriminated early- from late-mortality patients with strong prognostic performance (AUC up to 0.90), illustrating how advanced metagenomic analytics can extract clinically actionable signals from complex datasets to support patient-centered clinical management strategies in settings where rapid clinical deterioration is frequent (19).

A promising application of AI to metagenomics is represented by the ability to differentiate between viable and dead microorganisms, addressing a common challenge in modern microbiology where molecular positivity cannot distinguish live from dead organisms. In standardized laboratory conditions, Ürel et al. (20) developed a model capable of detecting the viability of bacteria with an accuracy of 0.83 and an AUC value of 0.90 through the analysis of shotgun nanopore sequencing data.

Yet, the promise of this panoramic vision must be tempered by current limitations. The perceived high costs of sequencing, coupled with the need for robust bioinformatic infrastructure and expertise, currently restrict metagenomics to high-stakes clinical scenarios, such as culture-negative infections (e.g., endocarditis), intraoperative diagnostics, or precision outbreak tracking.

However, it should be noted that in a 2021 cost-effectiveness simulation model of a 32-month carbapenem-resistant *A. baumannii* (CRAB) outbreak, early use of WGS and shotgun metagenomics was predicted to avert 18 additional infections, yield 74 extra quality-adjusted life years (QALYs), and reduce overall hospital expenditures by AU$93,822 compared with the real-life outbreak management scenario, highlighting the potential clinical and economic value of sequencing-guided infection control (21). Then, the integration into routine, high-throughput clinical microbiology should move from aspirational to practical.

## 5 AI-integrated sequencing for real-time genomic surveillance

During and after SARS-CoV-2-caused COVID-19 pandemic, pathogen WGS shifted from reduced capacity to surveillance backbone, enabling rapid lineage tracking, transmission mapping, and detection of emerging resistance across clinical and public-health settings. Consensus guidance now frames genomic surveillance as essential for infection control and AMR monitoring across One-Health domains (22).

The term “real-time” should not be interpreted as “instantaneous.” It should refer to a data flow that makes information timely for effective infection control decisions and public health policies. Real-time genomic surveillance should refer to the continuous process of monitoring and analyzing the genetic material of pathogens and AMR-associated distribution, as they circulate in a population, with the goal of providing actionable insights to control outbreaks as soon as possible as they occur.

Unlike traditional surveillance, which often relies on retrospective data, real-time surveillance should aim for a fast “sample-to-result” TAT, typically within days or weeks, to allow public health officials to intervene during an active transmission event rather than simply documenting it after the event.

Real-time genomic surveillance has been proposed to anticipate the emergence of resistant strains, track their spread, link human, animal, and environmental data, and support rapid response.

As partially anticipated, currently, two complementary sequencing paradigms are fundamental for surveillance: (i) isolate WGS, which delivers high-resolution phylogenetics and precise AMR determinant calling, and (ii) metagenomic NGS (mNGS), which profiles mixed specimens (e.g., blood, cerebrospinal fluid, bronchoalveolar lavage, wastewater) without culture.

A 7-year clinical mNGS evaluation highlighted stable diagnostic yield but also the operational drivers of TAT: 8.2–11.4 days from sample collection to result and 3.6–3.8 days from start of processing to result. It emphasizes the need for workflow engineering and automation that include the pre-analytical phase (decision to order testing, sample shipping, batch testing) to achieve true “real-time” (23). AI-assisted triage could be a practical target for improvement of the pre-analytics. Examples of AI-based systems applications include auto-eligibility of cases that might benefit from NGS (e.g., culture-negative samples), automated creation of samples shipping orders, and efficient batching of a sufficient number of specimens for NGS (24).

In 2021, in a study on new workflow for real-time prediction of pathogenic microorganisms, Bartoszewicz et al. (25) built DL models specialized in inference from incomplete short- and long-read sequencing data. The models showed an accuracy higher than 78.5% in inferring whether an Illumina or a Nanopore sequence belongs to pathogenic bacteria or viruses, for reads as short as 50 bp obtained during the Illumina sequencing run or after the first 250 bp (0.5 s of sequencing time) of Nanopore reads (25). If properly validated in the clinical context, such AI-based systems, performed in real-time during the sequencing run, could potentially save hours (depending on the platform used) in the post-analytical phase of pathogens identification.

However, Liu et al. (26) showed that adaptive nanopore sequencing, even without AI capabilities, already enabled reliable pathogen identification using the reads obtained within the first hour of sequencing, with a categorical agreement (CA) of 89.3%. Overall, it achieved 90.6% CA for AMR prediction with very major error rates of only 1–3% in key pathogens, and detected 23 polymicrobial infections, including 7 missed by standard diagnostics, substantially improving timely stewardship and outbreak control (26).

Beyond diagnostics, long read-based technologies like nanopore resolve complex AMR loci and structural variants that short-read pipelines may miss, strengthening surveillance signals about transmission of resistance cassettes (2).

Multiple evaluations now show that embedding WGS into infection-prevention programs improves timeliness and precision of outbreak recognition, with measurable patient and economic benefits when results are integrated into routine workflows. Optimized pipelines can move from sample to action in a clinically relevant window, particularly when coupled to automated analytics and visualization for infection prevention and control teams (27). This combination of AI and WGS has been successfully evaluated for outbreak investigation in hospital settings. Sundermann et al. (28) developed an AI algorithm that helped identify likely transmission routes after genomic clustering was established. The model increased the identification of epidemiological links by 3.8% as compared to traditional review methods of infection prevention and control, with an overall sensitivity of 91.7% (28).

For population-scale surveillance studies, including resistome surveillance, metagenomic analysis of sewage has been proposed as a powerful tool, capturing antibiotic resistance gene (ARG) diversity and trends independent of testing behavior. Large international datasets demonstrated global heterogeneity of ARGs and their association with socio-economic and environmental factors, while newer studies use serial wastewater metagenomes to relate resistome shifts to antimicrobial use and interventions, positioning wastewater-based epidemiology (WBE) as a sensitive barometer for public-health AMR policies (29, 30). WBE might be integrated with AI-based tools to improve surveillance capabilities. Liu et al. (31) demonstrate that ML models trained on WGS data using gene presence and absence profiles, SNP variants, and k-mer features can reliably predict antimicrobial resistance in *S. aureus*. In a dataset of 1,764 isolates tested against ten antibiotics, their best performing models achieved AUC values ranging from 0.83 to 0.99, with six antibiotics reaching an AUC above 0.99 and nine exceeding 0.96. These findings show that rich genomic feature sets enable ML classifiers to capture resistance determinants with consistent accuracy across various antimicrobial agents (31).

Emerging studies clarify that “real-time” also involves an efficient integration of sample logistics, run time, computation, expert review, and feedback into decisions. Reported clinical mNGS experiences indicate that shaving days off logistics and report generation often dwarfs gains from faster chemistry alone, areas where AI-driven automation (eligibility triage, templated narratives, anomaly alerts) can reduce wall-clock time (32).

## 6 Integration of artificial intelligence with other technologies

While this review centers on genome-based AI for AMR prediction, emerging non-genomic tools like Raman spectroscopy and matrix-assisted laser desorption/ionization-time of flight mass spectrometry (MALDI-TOF MS) can complement these approaches. When paired with ML, they offer rapid, label-free resistance profiling, especially useful when sequencing is unavailable or delayed.

### 6.1 Raman spectroscopy and machine learning

While culture-based methods remain the diagnostic standard, their prolonged TAT might be inappropriate for the urgency of severe infections. The integration of Raman spectroscopy with ML emerges as a powerful challenger, potentially poised to redefine rapid microbiological identification, before culture results are available (33, 34). Specimens from nasopharyngeal, rectal, or wound swabs are analyzed with minimal preparation, significantly shortening diagnostic timelines. The derived spectral patterns can then be fed to supervised ML classifiers, such as SVMs, Random Forests, and deep neural networks, which promptly identify not only microbial species, but also related AMR phenotypes (35). The single-cell resolution offered by Raman spectroscopy with ML, could represent a valuable tool for the diagnostics of pathogenic bacteria and AMR. The study by Lu et al. (36) showed an average accuracy in the identification of 12 species of common pathogenic bacteria (e.g., *E. coli*, *K. pneumoniae*, *S. aureus*) equal to 90.73 ± 9.72%. While the model built for the study was unable to discern specific antibiotic resistance, it was able to discriminate with 99.92 ± 0.06% accuracy drug-resistant *A. baumannii* isolates from susceptible clones. However, Raman spectroscopy has not yet been applied to pathogenic identification and antibiotic susceptibility detection in clinical practice, limiting the generalizability of these observations (36). Practical barriers include the need for standardized spectral databases, variability in sample preparation, model generalizability across diverse clinical settings, and limited interpretability of spectral features (37). While the approach shows clear promise, its adoption will depend on addressing technical, operational, and regulatory hurdles.

### 6.2 Mass spectrometry and machine learning

While MALDI-TOF MS has already revolutionized microbial identification, its integration with ML is now extending its capabilities into strain-level epidemiology and predictive resistance profiling. This synergy is particularly impactful for the direct analysis of clinical swabs, moving beyond mere identification to deliver actionable insights at a pace that could directly influence patient outcomes. The performance of any downstream algorithm is tightly coupled to the quality and diversity of the reference spectral datasets. The added value of the integration of ML with MALDI-TOF becomes evident precisely where traditional methods reach their limits. For example, complex taxonomic groups like the *Enterobacter cloacae* complex often confuse conventional diagnostics, whereas MALDI-TOF combined with ML can accurately carry out species-level discrimination tasks (38). Perhaps more significantly, it can be scaled to outbreak investigations, differentiating epidemiologically relevant strains (e.g., *P. aeruginosa* ST175) when WGS is not available, promptly informing infection control teams (39). For common pathogens like *Klebsiella* spp. or *E. coli*, the challenge is no longer identification, but rapid resistance profiling since predicting antibiotic resistance within hours rather than days allows a swift transition from empirical to targeted therapy. Ultimately, ML does not merely automate MALDI-TOF interpretation; but transforms a gold-standard identification tool into a dynamic source of high-fidelity intelligence for clinical decision-making as shown in several recent studies. In one example, a 2024 investigation on *Staphylococcus epidermidis*, through the analysis of best-annotated protein, reported 94% accuracy in predicting methicillin resistance based on subtle spectral variations, circumventing the 24-h delay associated with traditional testing (40). Comparably, in Gram-negative rods a neural network trained to identify carbapenem-resistant *Klebsiella pneumoniae* showed over 90% accuracy (41). The combination of ML and MALDI-TOF marks a pivotal shift in AMR diagnostics. Although the technological potential is undeniable, bringing these innovations into daily clinical use reveals a familiar tension: progress in research outpacing its clinical integration. In fact, high performance often depends on narrowly selected datasets, while real-world clinical samples are often polymicrobial infections and with poor-quality spectra. Additionally, many ML-derived spectral features lack clear biological annotation. It is one thing to identify a peak statistically associated with resistance; it is another to link it mechanistically without confirmatory genomic evidence, a step frequently skipped. Regulatory concerns further complicated the matter. Still, the momentum behind this technology should not be lost. Addressing these issues is not just desirable but needed, if we aspire to move from proof-of-concept to practical, trustworthy tools in patient care.

## 7 State of the art and current challenges

### 7.1 State of the art

The integration of AI into clinical microbiology might represent a valuable transition from culture-dependent diagnostics to proactive, data-driven decision-making. The convergence of genome sequencing and other technologies is delivering significant improvements in speed, accuracy, and diagnostic resolution (33, 38). One of the most significant benefits would lie in the ability to potentially reduce the number of hours imposed by the standard diagnostic timeline, allowing clinicians to act earlier and with greater precision. This emerging diagnostic landscape, summarized in Table 2, also promises to alleviate the heavy manual workload in clinical microbiology laboratories, automating time-consuming tasks, and allowing skilled personnel to focus on higher-order analytical and interpretive challenges. The field of AI-assisted microbiology against AMR is growing rapidly, with a diverse array of models and modalities emerging to address different clinical and public health needs. While this variety reflects innovation and potential, it can be overwhelming for clinicians and decision-makers. Many published models are trained in tightly scoped settings, optimized for specific data types or institutions, and reported using performance metrics that may not generalize beyond their original context. The result is a proliferation of tools whose applicability, reproducibility, and true clinical impact are not always clear. Publication bias further complicates interpretation, as models are often internally tuned and validated on the same data until optimal results are achieved, potentially giving a misleading impression of robustness. Looking at common failure modes that may limit model performance or generalizability in real-life scenarios, we found (1) the risk of dataset shifts, for both geographical and methodological reasons, to be relevant as many studies were analyzing data coming from publicly available microbiology datasets collecting isolates’ data from all over the world, and often the geographic origin of the isolate, or methodological differences in isolates’ analyses, were not taken into account (8, 10, 11, 25, 30), other than in the studies by Munk et al. (29) and Candela et al. (38). Moreover, (2) only two studies mentioned training the model on one dataset and then testing on other datasets pursuing multicenter external validation (38, 40), whereas many experiments relied on hold-out validation or internal train/test splits (9, 20, 25, 35, 41); or pursued k-fold cross validation (10, 11, 32, 36). Thirdly, (3) Only a minority explicitly addressed the risk of phylogenetic confounding (10, 20, 40), for example by analyzing DNA degradation signatures, despite evidence that some models may be capturing and learning lineage-related features (e.g., k-mer counts or taxonomic features), rather than detecting causal resistance determinants mechanisms (9, 11, 25, 31, 35, 36, 41). Furthermore, (4) clinical interpretability of findings remains uneven among studies reporting on antimicrobial susceptibility as only one study framed errors in terms of major and very major rates (9), while the vast majority reported only global metrics such as accuracy or AUROC and precision-recall curves, sensitivity, and specificity, without extensively addressing misclassification risk, which obscure risks relevant to frontline use in different scenarios (10, 11, 20, 31, 35, 36, 40, 41). Finally, (5) transparency also varied but there are encouraging signals worth mentioning: several included studies shared public access to the reference code on platforms such as GitHub, or released the model under an open license (9, 11, 20, 25). Moreover, 6 papers are directly referencing publicly available microbiology datasets (e.g., NCBI Sequence Read Archive, PATRIC ID, DRIAMS) (8, 10, 19, 29, 38); only one study guaranteed both full microbiological data and code availability (40). This shows an effort towards reproducibility is being made, which is a fundamental step towards broader adoption and future multicenter validation of these models. Taken together, these findings underscore the importance of rigorous validation and full-spectrum risk reporting before deployment of AI tools into clinical microbiology and surveillance workflows.

**Table 2:** Summary of included studies describing AI task and models, input data, examples among included studies, and public-health applications.

Core model task	Main clinical/lab application	Input data types	AI approaches	Example in paper	Public health use case
Pathogen or taxon identification and characterization	Rapid species identification	MALDI-TOF MS spectra	RF; SVM; PLS-DA; PCA	*E. cloacae complex* species discrimination vs. reference databases (38)	Outbreak-relevant taxonomy
	Rapid pathogen identification	Raw NGS reads (Illumina/Nanopore)	CNN; LSTM	Real-time DL classification of pathogenic reads during sequencing (25)	Biosecurity surveillance
	Rapid pathogen identification	Single-cell Raman spectra	CNN compared with RF; SVM; AdaBoost; RF + PCA	Single-cell Raman → RF pathogen identification and AST (35, 36)	Early AST-guided public health response
	Viability classification	Raw nanopore signal time-series	CNN; XAI (Class activation maps)	DL-based viability inference with explainable “signal-drop” patterns in metagenomics (20)	Explainable AI application in fast microbiology; Antimicrobial and diagnostic stewardship
Outbreak detection and clone chatacterization	Rapid high-risk clone detection	ALDI-TOF MS; FTIR-S spectra; WGS data	RF; SVM; NCA-kNN; PLS-DA; PCA	Identification of a high-risk clone (GC1- vs. non-GC1) via genome sequencing (11) Threshold vs. biomarker peak-matrix discrimination of a high-risk clone via MALDI-TOF (39)	Outbreak strain surveillance
	Hospital outbreak investigation support + transmission-route attribution	WGS data + EHR spatiotemporal data	Logistic regression; likelihood-ratio ML; importance sampling	EDS-HAT: WGS–EHR integration identifies hidden outbreaks and routes (27, 28)	Improved IPC containment; population data mining for AMR surveillance
Discovery of Resistance determinants and resistome structure	Resistome profiling	Shotgun metagenomics; antibiotic resistance genes profiles	DL; Kraken2; PCA; PERMANOVA; networks	Plasmid detection + mapping of global resistome (29); framing and interpretation of resistome structure and antibiotic usage associations (30)	Global AMR surveillance (One Health perspective); early-warning systems
	Resistance determinant discovery	Public genomes (PATRIC); WGS assemblies; pan-genome gene/allele matrices	RF; SVM; NB; AdaBoost; GA feature selection	Pan-genome SVM ranking identifies genes potentially involved in new mechanisms of resistance (8, 10)	Advancement in AMR genotype knowledge
	ICU resistome analysis for clinical risk stratification purposes	Clinical variables; shotgun metagenomics (from patient and environmental sampling); distance matrices	ML-adjacent methods (PCA, network inference)	ROC/AUC-based regression plus multivariate ordination and network analysis (19)	Population-level pattern discovery
Antimicrobial susceptibility prediction	Binary AMR classification	WGS assemblies; pan-genomic library (gene presence/absence)	RF; SVM; NB; AdaBoost; GA feature selection	Pan-genome workflow identifying known AMR determinants (8, 10)	Improved IPC containment Enhanced antimicrobial stewardship
	Binary AMR classification	MALDI-TOF MS spectra; +/− AST labels	RF; SVM; LR; NB; LightGBM; MLP; SHAP; ANN + feature selection	RF-mediated interpretable AMR predictions (40) Full-spectrum ANN CRKP screening before AST (41)	Improved IPC containment; Enhanced antimicrobial stewardship; Explainable AI-based AMR surveillance
	Accelerated identification and AST	Single-cell Raman spectra	CNN compared with RF; SVM; AdaBoost; RF + PCA	Culture free (Raman spectroscopy + RF) pathogen identification and AST (35, 36)	Early AST-guided public health response
	MIC prediction → S/I/R classification	WGS reads; k-mer matrices	RF; SVM; XGBoost;	k-mer–based MIC prediction capturing *S. aureus* resistance mechanism (SCCmec) and phenotypic resistance prediction (9)	Improved IPC containment; Enhanced antimicrobial stewardship

AI, Artificial Intelligence,; ML, Machine Learning; AMR, Antimicrobial Resistance; AST, Antimicrobial Susceptibility Testing; IPC, Infection Prevention and Control; ROC, Receiver Operator Characteristic curve; AUC, Area Under the Curve; SVM, Support Vector Machine; DL, Deep Learning; kNN, k-Nearest Neighbour; CNN, Convolutional Neural Network; ANN, Artificial Neural Network; DT, Decision Trees; RF, Random Forest; NB, Naïve Bayes; AdaBoost, Adaptive Boosting; XGBoost, Extreme Gradient Boosting; LightGBM, Light Gradient Boosting Machine; PCA, Principal Component Analysis; SHAP, SHapley Additive exPlainations; FTIR-S, Fourier-transform Infrared Spectroscopy; MALDI-TOF MS, Matrix-Assisted Laser Desorption/Ionization Time-of-Flight Mass Spectrometry; LR, Logistic Regression; GA, Genetic Algorithm; XAI, eXplainable AI; PERMANOVA, Permutational Multivariate Analysis of Variance; PLS-DA, Partial Least Squares Discriminant Analysis; CRKP, Carbapenem-resistant *Klebsiella pneumoniae*; CSKP, Carbapenem-susceptbile *Klebsiella pneumoniae*; LSTM, Long Short-Term Memory, NGS, Next Generation Sequencing; EDS-HAT, Enhanced Detection System for Healthcare-Associated Transmission; EHR, Electronic Health Record; MLP, Multilevel Perceptron.

### 7.2 Current challenges

Several critical obstacles, some of which have already been mentioned above, must be addressed before these technologies can be adopted widely and safely. First, the cost of acquiring and implementing AI-integrated platforms, especially those requiring advanced sequencing or high-resolution imaging, is a major barrier for many institutions. Second, the performance and fairness of AI systems are directly tied to the quality and diversity of their training data. As Peiffer-Smadja et al. (34) highlight, the lack of large, standardized, and globally representative datasets, particularly for spectral and genomic data, limits model generalizability and raises the risk of diagnostic bias across different populations and healthcare settings. Third, the so-called “black box” nature of many DL models continues to undermine clinical trust. Clinicians are understandably reluctant to rely on predictions that lack transparent reasoning, especially in high-stakes decisions. Fourth, the lack of reproducibility requirements and external validation that result in lack of generalizability. Finally, such AI-based systems should be integrated, as appropriate, with specific resources like databases, that collect and organize reference information on AMR, and international guidelines to interpret AMR (42, 43).

To advance, the field must commit to addressing these challenges through open, interdisciplinary collaboration. Key actionable priorities could include: (a) developing expansive, curated, and ethically sourced multimodal databases that include diverse patient populations and microbial clones, (b) improving explainable AI methodologies that make model outputs interpretable and auditable (e.g., enabling clinicians to visualize which specific spectral peaks or resistance genes underpin a given prediction), (c) designing hybrid, tiered diagnostic systems that combine rapid techniques (e.g., Raman spectroscopy) with high-resolution methods (e.g., metagenomic sequencing) to create scalable workflows tailored to clinical urgency and case complexity, and (d) ensuring the development and availability of cost-effective and accessible technologies across diverse healthcare settings to mitigate disparities in AI adoption and prevent technology-driven discrimination, (e) fostering prospective, multicentric validation of AI tools to explore real-world usability and understand how AI tools behave under heterogeneous epidemiology, laboratory practices, patient case-mix, and data quality to support safer deployment and more appropriate scope definition before widespread adoption, and (f) encouraging the use of AI agent-based coordination approaches to integrate outputs from multiple task-specific tools, with the aim of reducing information fragmentation and supporting coherent, prioritized clinical decision-making in complex clinical workflows.

By addressing these limitations head-on, the fusion of AI and microbial diagnostics can evolve from isolated innovations into a robust, intelligent, and equitable diagnostic ecosystem, one that accelerates results and enhances precision, adaptability, and resilience in clinical microbiology.

## 8 Relevance for public health and future perspectives

The methodologies reviewed in this paper, specifically the application of AI to genomic strategies, offer transformative opportunities for pathogen identification and AMR detection. These AI-driven tools hold the potential to substantially reduce diagnostic TAT, enabling clinicians to initiate targeted therapies with greater promptness and appropriateness. As a result, the implications of AI-aided sequencing and spectroscopy for public health are profound: at the bedside, ML models applied to MALDI-TOF or Raman spectra can deliver rapid AMR predictions, offering valuable support for early treatment decisions and antimicrobial stewardship. Further upstream, WGS and mNGS provide higher-resolution insights, enabling the detection of transmission clusters, characterization of resistance mechanisms, and tracking of high-risk clones across healthcare facilities and regions. These genomic approaches may also underpin sentinel surveillance programs and broader population-level initiatives, such as national strain monitoring and prevalence mapping, though they require robust infrastructure, standardized workflows, and centralized coordination. Wastewater-based epidemiology is emerging as a complementary strategy, leveraging mNGS to detect shifts in community-level resistomes and early signs of emerging threats, though analytical complexity remains a barrier to widespread implementation. In low-resource settings, simplified and portable solutions, such as real-time nanopore sequencing or MALDI-based species identification, can provide actionable insights with lower technical requirements, provided there is investment in training and system integration.

The near future likely lies in a tiered diagnostic framework, which may help balance speed, cost, and resolution: for instance, deploying MALDI-TOF or Raman-based ML for rapid, priority triage, while reserving WGS or mNGS for high-risk outbreak scenarios, such as MDRO outbreaks in large ICUs or oncohematological facilities, that require deeper genomic resolution and contextual interpretation. Moving forward, the integration of population-based metrics other than hospital systems’ EHRs may warrant future integration of AI in public health interventions against AMR such as improving vaccine uptake in fragile populations or assist and monitor the prescription of antimicrobials in primary care (44, 45).

Such a stratified approach balances diagnostic depth with operational feasibility, ensuring that high-resolution insight is applied precisely where it is needed most.

## 9 Conclusion

Taken together, the evidence reviewed indicates that AI has the potential to substantially enhance clinical microbiology and AMR surveillance, but its impact will depend on disciplined translation rather than technological novelty alone. Progress will require robust validation, transparent reporting of clinically meaningful errors, and seamless integration of AI outputs into resource-aware and coherent diagnostic workflows. Only through such a rigorous and pragmatic path can AI move from isolated proofs of concept to reliable components of routine microbiological practice.
