# Correction: Integrating artificial intelligence with genome sequencing against antimicrobial resistance: a narrative review

**DOI:** 10.3389/fpubh.2026.1824508

**Published:** 2026-04-14

**Authors:** Giovanni Scaglione, Nicolò Mastroianni, Alberto Rizzo, Emanuele Palomba, Davide Carcione, Sara Giordana Rimoldi, Gioconda Brigante, Luigi Principe, Marta Colaneri, Andrea Gori, Fabio Borgonovo

**Affiliations:** 1 Department of Infectious Diseases, Luigi Sacco Hospital, Milan, Italy; 2 Laboratory of Clinical Microbiology and Virology, ASST Valle Olona, Gallarate, Italy; 3 Laboratory of Clinical Microbiology, Virology and Bioemergencies, Luigi Sacco Hospital, Milan, Italy; 4 Centre for Multidisciplinary Research in Health Science (MACH), University of Milan, Milan, Italy; 5 Clinical Microbiology and Virology Unit, Great Metropolitan Hospital “Bianchi-Melacrino-Morelli”, Reggio Calabria, Italy; 6 Department of Biomedical and Clinical Sciences “L. Sacco”, University of Milan, Milan, Italy

**Keywords:** antimicrobial resistance, artificial intelligence, diagnosis, genome sequencing, infection, machine learning, surveillance

Author Sara Giordana Rimoldi was omitted as an author in the published article. The correct author list reads:

Giovanni Scaglione^1^, Nicolò Mastroianni^2^, Alberto Rizzo^3^^*^, Emanuele Palomba^1, 4^, Davide Carcione^2^, Sara Giordana Rimoldi^3^, Gioconda Brigante^2^, Luigi Principe^5^, Marta Colaneri^4, 6^, Andrea Gori^1, 4, 6^, Fabio Borgonovo^1^

^1^Department of Infectious Diseases, Luigi Sacco Hospital, Milan, Italy, ^2^Laboratory of Clinical Microbiology and Virology, ASST Valle Olona, Gallarate, Italy, ^3^Laboratory of Clinical Microbiology, Virology and Bioemergencies, Luigi Sacco Hospital, Milan, Italy, ^4^Centre for Multidisciplinary Research in Health Science (MACH), University of Milan, Milan, Italy, ^5^Clinical Microbiology and Virology Unit, Great Metropolitan Hospital “Bianchi-Melacrino-Morelli”, Reggio Calabria, Italy, ^6^Department of Biomedical and Clinical Sciences “L. Sacco”, University of Milan, Milan, Italy

The **Author Contributions** Statement has been corrected to read:

GS: Conceptualization, Writing – original draft, Writing – review & editing, Supervision. NM: Writing – original draft, Writing – review & editing. AR: Supervision, Writing – review & editing. EP: Supervision, Writing – review & editing. DC: Writing – review & editing. SR: Resources, Writing – review & editing. GB: Writing – review & editing. LP: Writing – review & editing. MC: Supervision, Writing – review & editing. AG: Funding acquisition, Resources, Writing – review & editing. FB: Conceptualization, Writing – original draft, Writing – review & editing, Supervision.

The original version of this article has been updated.

