# Supplementary material for: Integrating artificial intelligence with genome sequencing against antimicrobial resistance: a narrative review
Source: Front Public Health. 2026 Jan 29;14:1757161. doi: 10.3389/fpubh.2026.1757161 (PMC12894283; doi:10.3389/fpubh.2026.1757161)
Supplement: Supplementary file 1 [file Table_1.DOCX]

**Supplementary Table 1. PICO framework.**

| **Element** | **Description** |
| --- | --- |
| **P – Population** | \| Hospitalized patients, healthcare-associated infections, microbiology laboratory workflows for detection, surveillance, and control of antimicrobial-resistant pathogens. \| \| --- \|   *Keywords:* "Hospitals", "Inpatients", "Healthcare Facilities", "Nosocomial Infections",, "MRSA", "VRE", "CRE", “CRAB”, “CRPA”, "Multidrug-Resistant Organisms", “Surveillance”, “Nosocomial infections”, “Clinical microbiology”, “Rapid diagnostic technologies”, “Fast microbiology”, “Whole-genome sequencing”, “Next-generation sequencing”, “Microbiological surveillance”, “Culture-based methods”, “Metagenomics”, “Nanopore sequencing”, “MALDI-TOF MS”, “Raman spectroscopy”, “AI-WGS integration”, “Real-time surveillance”, “Clinical microbiology automation”. |
| **I – Intervention** | Artificial intelligence-based methods applied to genomic, metagenomic, and rapid phenotypic microbiology data to support antimicrobial resistance detection, prediction, surveillance, and infection control.  Interventions include machine learning and deep learning models integrated with WGS, mNGS, MALDI-TOF MS, Raman spectroscopy, and sequencing workflows, used for resistance prediction, transmission inference, outbreak investigation, laboratory automation, and decision support.  *Keywords:* "Artificial Intelligence", "Machine Learning", "Deep Learning", "Natural Language Processing", “Convolutional Neural Network”, "Predictive Analytics", "Infection Control Decision Support", “protein language models”, “rule-based systems”, “CDSS”. |
| **C – Comparison** | *Not applicable.* This review is focused on mapping current AI applications; no comparator is required for inclusion. |
| **O – Outcomes** | 1. Diagnostic and analytical performance   - Pathogen identification   - Genotypic and phenotypic AMR prediction   - Resistome and virulome characterization 2. Operational and workflow impact   - Turnaround time reduction   - Automation of microbiology laboratory processes   - Scalability and real-time usability 3. Surveillance and infection control relevance   - Detection of transmission chains   - Outbreak investigation   - Support for infection prevention and control strategies 4. Implementation and translational challenges   - Data integration and standardization   - Model generalizability and bias   - Interpretability, regulatory, and cost-related issues     *Keywords:* “Pathogen detection”, “antimicrobial-resistance detection”, "Hospital Infection Control", "Outbreak Detection", "Implementation Science", "Program Evaluation", "Performance Monitoring", "Real-Time Surveillance", “Resistance Prediction”, “Clinical decision support”, “Bias Mitigation”. |
